# Age-Related Variations in Clinical, Histological, and Genetic Characteristics in Multiple and Familial Melanomas: A Study of 333 Patients

**DOI:** 10.3390/jcm14030686

**Published:** 2025-01-22

**Authors:** Andrea Carugno, Giovanni Paolino, Mario Valenti, Noemi Brigenti, Lorenza Bertù, Andrea Gianatti, Paolo Sena, William Bruno, Paola Ghiorzo, Fabio Pagni, Nicola Zerbinati

**Affiliations:** 1 Department of Medicine and Surgery, University of Insubria, 21100 Varese, Italy; lorenza.bertu@uninsubria.it; 2 Dermatology Unit, Ospedale di Circolo Fondazione Macchi, ASST Sette Laghi, 21100 Varese, Italy; nicola.zerbinati@uninsubria.it; 3 Ph.D. Program in Molecular and Translational Medicine (DIMET), University of Milan-Bicocca, 20126 Milan, Italy; fabio.pagni@unimib.it; 4 Unit of Dermatology, IRCCS Ospedale San Raffaele, 20132 Milan, Italy; paolino.giovanni@hsr.it; 5 Faculty of Medicine and Surgery, Università Vita-Salute San Raffaele, 20132 Milan, Italy; 6 Dermatology Unit, IRCCS Humanitas Research Hospital, 20089 Rozzano, Italy; mario.valenti@hunimed.eu; 7 Department of Biomedical Sciences, Humanitas University, Pieve Emanuele, 20089 Milan, Italy; 8 Section of Dermatology and Venereology, Department of Medicine, University of Verona, 37126 Verona, Italy; noemi.brigenti@studenti.univr.it; 9 Pathology Unit, ASST Papa Giovanni XXIII, 24127 Bergamo, Italy; agianatti@asst-pg23.it; 10 Dermatology Unit, ASST Papa Giovanni XXIII, 24127 Bergamo, Italy; psena@asst-pg23.it; 11 Cancer Genetics, IRCCS Ospedale Policlinico San Martino, 16132 Genoa, Italy; william.bruno@unige.it (W.B.); paola.ghiorzo@unige.it (P.G.); 12 Department of Internal Medicine and Medical Specialties, University of Genova, 16132 Genoa, Italy; 13 Department of Medicine and Surgery, Pathology, Fondazione IRCCS San Gerardo dei Tintori, 20900 Monza, Italy; 14 Department of Medicine and Innovation Technology (DiMIT), University of Insubria, 21100 Varese, Italy

**Keywords:** melanoma, multiple primary melanoma, familiar melanoma, genetic, risk factors, *CDKN2A*

## Abstract

**Background/Objectives**: Melanoma is an aggressive cutaneous malignancy with a rising incidence. While most cases are sporadic, 5–10% are hereditary, especially in patients with multiple or familial melanomas. The aim of this study is to explore the epidemiological, clinical, histological, and genetic features of this class of patients to identify risk factors for better management and surveillance. **Methods**: Between 2021 and 2024, patients with multiple melanomas or a familial history of melanoma were recruited. Collected data included demographic, clinic-pathologic features, and genetic analyses. **Results**: Patients >60 years had a higher prevalence of multiple melanomas (>50%, *p* = 0.0002), while familial melanoma was more common in those <40 years (54.3%). UV exposure increased with age, while sunscreen use decreased (*p* = 0.0004). Younger patients showed the highest nevi counts (mean: 139.6) and density (*p* < 0.0001). Dermatologists more frequently detected subsequent melanomas in older patients (>60 years) (*p* = 0.001). Genetic testing and melanoma subtypes showed no significant age-related differences. **Conclusions**: melanoma can develop at any age, and early detection through regular screening is crucial. Older patients (>60 years) have a higher prevalence of multiple melanomas, influenced by UV exposure and genetics. Indeed, in our cohort, a history of sun exposure, sunburns, and tanning bed use emerged as key risk factors, particularly among older individuals. Genetic testing showed a 4.3% rate of pathogenic/likely pathogenic variants, mainly in *CDKN2A*. Family history and nevus burden are significant risk factors, highlighting the need for targeted surveillance in high-risk populations.

## 1. Introduction

Melanoma is a type of skin cancer that originates from melanocytes, the cells responsible for producing melanin. Although melanoma typically arises on the skin, it can occasionally occur in other anatomical locations, such as mucous membranes, including the sinuses, nasal passages, oral cavity, vagina, anus, and rectum [1,2].

Surgical excision is highly effective for early-stage melanoma. However, systemic therapies are often required in advanced stages to control disease progression and improve patient outcomes. Despite these treatments, the five-year survival rate for metastatic melanoma remains low, highlighting the aggressive nature of this malignancy. In 2015 more than 350,000 new melanoma cases were diagnosed worldwide, corresponding to an age-standardized incidence rate of 5 per 100,000 individuals, with nearly 60,000 deaths attributed to the disease [3].

The incidence of cutaneous melanoma has been rising at an annual rate of 3% to 7%, likely driven by a combination of genuine increases in disease occurrence and advancements in diagnostic capabilities. Improved screening, higher biopsy rates, and greater histopathological accuracy have facilitated earlier detection, particularly of thinner melanomas [4]. For example, in Germany, the median tumor thickness has steadily decreased from 1.81 mm in the 1980s to 0.53 mm in 2000, accompanied by an increased diagnosis of in situ and thin melanomas (≤0.8 mm Breslow thickness). However, data from the United States indicate that incidence rates have risen across all tumor thickness categories, suggesting that enhanced detection alone cannot fully account for this overall trend [5,6].

Vertical tumor thickness, or Breslow depth, is the most critical prognostic factor for primary cutaneous melanoma, with greater thickness correlating with an increased risk of adverse outcomes. The prevalence of thick melanomas rises with age, reaching approximately 20% in individuals aged 80 years or older. Additional prognostic factors, such as ulceration, lymphatic invasion, and host immune response, also influence disease severity and guide therapeutic strategies. Early intervention is essential for improving survival, particularly in high-risk stages (II–IV), where adjuvant therapies, including immune checkpoint inhibitors and targeted agents (e.g., BRAF and MEK inhibitors), are crucial for delaying disease progression and extending survival [5,6].

Melanoma is the second most common cancer among individuals aged 0 to 39 years, a demographic categorized as Adolescents and Young Adults (AYAs) [7,8,9,10]. The rising incidence in this group is primarily driven by environmental and behavioral factors, including the use of indoor tanning devices, which increase melanoma risk by 75% when used before the age of 30. Furthermore, intermittent UV exposure without adequate protection is a significant contributor to melanoma development in AYAs. Advances in diagnostic techniques, particularly dermoscopy, have allowed for earlier detection in this population [7,8,9,10].

Despite the rising prevalence of melanoma among AYAs, research specifically addressing this cohort remains limited. Existing studies reveal inconsistencies in clinical presentation, prognosis, and survival outcomes when comparing AYAs with older adults. For example, some evidence indicates that melanomas in AYAs display more aggressive characteristics, such as greater Breslow thickness and more advanced stages at diagnosis. However, other studies report better survival rates in younger patients, even when aggressive tumor features are present [7,8,9,10]. On a genetic level, melanomas in AYAs frequently harbor mutations in the *BRAF* gene, observed in 40–60% of cases, whereas older patients more commonly exhibit mutations in *NRAS*, *NF1*, or *TP53*, which are associated with chronic sun exposure [1,11].

Germline pathogenic variants can predispose individuals to melanoma, although inherited genetic susceptibility accounts for only a small proportion of cases. Approximately 5–10% of melanomas occur in individuals with a positive family history, but familial clustering is not always due to the inheritance of a single pathogenic variant. The genetic landscape of melanoma includes rare high-penetrance genes, such as *CDKN2A*, which are implicated in some familial cases, as well as more common pigmentation-related genes, such as *MC1R*, that increase the susceptibility of fair-skinned individuals to melanoma [12,13].

*CDKN2A* is the primary high-penetrance gene associated with familial melanoma, accounting for approximately 2% of all cutaneous melanoma cases. It encodes two distinct proteins, p16 and p14ARF, produced via an alternative reading frame. These proteins are essential for regulating cell cycle progression through the retinoblastoma protein (Rb) and p53 tumor suppressor pathways, respectively. Germline *CDKN2A* variants are identified in 20–40% of familial melanoma cases, with mutation penetrance varying widely (5–70%) across populations. This variability is influenced by environmental factors and genetic modifiers, such as *MC1R* variants. Furthermore, carriers of *CDKN2A* variants have an increased risk of developing pancreatic cancer, highlighting the broader oncological implications of these mutations [13,14,15,16,17].

Less common but significant high-risk genes include *CDK4*, a critical regulator of the cell cycle; *BAP1* (*BRCA1*-associated protein 1), associated with *BAP1* tumor predisposition syndrome; and telomere maintenance genes, such as *TERT* and *POT1* [12]. Pathogenic variants in these genes not only increase the risk of cutaneous melanoma but also predispose individuals to other malignancies. Although the spectrum of associated cancers is not yet fully understood, the growing recognition of melanoma-cancer syndromes underscores the need for tailored surveillance and management strategies for affected individuals [12].

A subset of patients with inherited variants exhibits a higher propensity for developing multiple primary melanomas (MPMs), defined as two or more distinct melanomas occurring in the same individual. MPMs are more common in individuals with *CDKN2A* or *CDK4* variants and typically present at a younger age compared to sporadic melanomas. These melanomas are often associated with a more aggressive clinical course, highlighting the critical importance of rigorous dermatological surveillance [1,11].

The genetic heterogeneity of melanoma, ranging from high-penetrance familial variants to polygenic influences on pigmentation and UV sensitivity, highlights the complexity of its pathogenesis. Early genetic screening, particularly in individuals with a family history of melanoma or features suggestive of an inherited predisposition, is critical for identifying patients at risk, guiding personalized surveillance strategies, and improving outcomes. Despite advances in our understanding, the interaction between genetic predisposition and environmental factors remains an ongoing area of research, offering opportunities to refine preventive and therapeutic approaches [18].

Understanding the unique biological and clinical characteristics of both young and older melanoma patients is essential for developing age-specific treatment strategies and improving long-term outcomes in this underserved population. By focusing on patients with multiple or familial melanoma, this research aims to elucidate the interplay between age, genetic predisposition, and clinical outcomes, ultimately contributing to the development of tailored treatment strategies and improved prognostic models for melanoma patients across all age groups.

## 2. Materials and Methods

This retrospective observational study was conducted to analyze the clinical and genetic data of melanoma patients followed from 2021 to 2024 at ASST Papa Giovanni XXIII Hospital in Bergamo, Italy. The study focused on individuals with multiple melanomas (two or more distinct primary melanomas) or a family history of melanoma, defined as having at least one second-degree relative with the disease. Special attention was given to identifying patients with risk factors for melanoma susceptibility, including genetic variants associated with the condition. The study was approved by the Ethics Committee of Bergamo under resolution n°834, dated 24 May 2022. It adhered to the principles outlined in the Helsinki Declaration, ensuring that the research was conducted ethically. All patients provided informed consent for their anonymized data to be used for scientific purposes.

A detailed clinical record was created for each patient after informed consent was obtained. Data collection covered multiple domains to ensure a thorough analysis; demographic information included age, sex, place of birth, place of residence, weight, height, body mass index (BMI), education level, marital status, and occupation. Phenotypic characteristics were documented, including skin phototype based on the Fitzpatrick scale, eye color, and natural hair color at age 20. Sun exposure habits were carefully assessed, including personal history of sun exposure, childhood sunburns, sunscreen use, tanning bed use, and occupational sun exposure. Family history of melanoma was recorded in detail, including the presence of affected relatives, degree of relationship, age at diagnosis, and any personal or family history of melanoma-associated cancers, such as kidney, pancreatic, or serous tumors. Data on known genetic variants predisposing to melanoma were included when available.

Genetic testing was performed in collaboration with the Cancer Genetics Unit in Genoa, Italy, with support from the Italian Melanoma Intergroup (IMI). Germline genetic analysis was performed using next-generation sequencing (NGS) with a panel focused on key melanoma susceptibility genes, including *CDKN2A*, *CDK4*, *BAP1*, *POT1*, *ACD*, *TERF2IP*, and *MITF*. Our center adopted the IMI inclusion criteria for eligibility for genetic testing [19]: (1) Diagnosis of cutaneous melanoma with a positive family history, defined as at least two affected relatives within the same family branch, with at least one diagnosed before the age of 60. (2) Personal history of multiple cutaneous melanomas, with at least two diagnoses, the first occurring before the age of 60. (3) Personal or family history of cutaneous melanoma associated with at least one of the following malignancies: pancreatic adenocarcinoma, uveal melanoma, pleural or peritoneal mesothelioma, renal neoplasms, or BAP1-inactivated melanocytic tumors, with a total of at least two oncologic diagnoses.

The genetic variants identified in this study were classified according to the most recent international guidelines for melanoma susceptibility. For the purpose of this analysis, only variants classified as pathogenic (class 5, “Very high likelihood that the variant is causative of the disorder”) and likely pathogenic (class 4, “High likelihood that the variant is causative of the disorder”) variants were considered [20,21]. Variants classified as benign or of uncertain significance were excluded from the study.

Clinical and histopathologic data were systematically collected for each melanoma diagnosis. This included melanoma location, age at diagnosis, clinical presentation, and histopathologic features. Key parameters recorded included histotype, Breslow thickness, Clark level, ulceration, regression, radial and vertical growth phases, mitotic count, nevus-associated melanoma, sentinel lymph node status, and the presence of loco-regional or systemic nodal involvement and visceral metastases. TNM classification was also documented. For patients with multiple melanomas, each additional melanoma was confirmed by histopathological diagnosis of primary lesions. Patients with melanoma recurrences or cutaneous metastases were excluded from the study.

### Statistical Analysis

Data analysis was performed on the entire cohort of patients enrolled in the study. Descriptive statistics were used to summarize the data with stratification based on melanoma type, number, disease severity, and patient age. Categorical variables were summarized using contingency tables, presenting both absolute and relative frequencies. Continuous variables were described using standard sampling statistics, including mean, standard deviation, median, and minimum and maximum values.

Associations between clinical and histopathologic variables were evaluated using appropriate statistical tests. For continuous variables, the F-test was used to assess group differences, whereas categorical variables were compared using either the chi-squared test or Fisher’s exact test, depending on the expected frequencies in the contingency tables. All statistical analyses were performed using standard software, and a *p*-value of less than 0.05 was considered statistically significant. Data analysis was performed using SAS ODA (version 9.4, SAS Institute Inc., Cary, NC, USA).

## 3. Results

Out of 1305 melanoma patients followed in our tertiary center, a total of 333 (25.5%) were selected to meet the inclusion criteria for multiple or familial melanoma. The cohort showed a slight predominance of females (54.9%) compared to males (45.1%) (Table 1).

**Figure 1:**
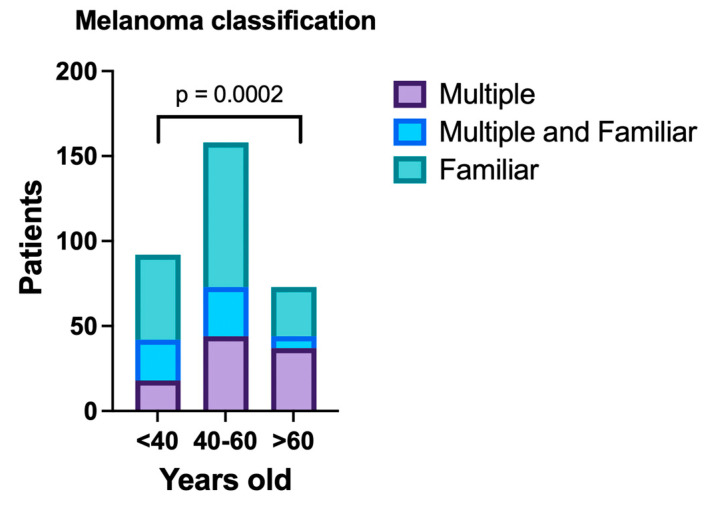
Distribution of melanoma subtypes across age groups. The classification includes multiple melanomas (purple), familial melanomas (green), and combined multiple and familial melanomas (blue). Patients over 60 years of age exhibited a significantly higher prevalence of multiple melanomas compared to younger age groups, with more than half presenting with multiple lesions. In contrast, familial melanoma was more common among younger patients, with 54.3% of individuals under 40 years showing a familial pattern (*p* = 0.0002).

Of the 333 patients enrolled, 49.2% were classified as having familial melanoma, 31.8% had multiple melanoma, and 18.9% had both multiple and familial melanoma (Figure 1). The mean age at first diagnosis of melanoma was 48.5 years. Phenotypically, all patients were of Caucasian descent, with the majority (58.2%) classified as having Fitzpatrick skin types I or II, which indicates a higher susceptibility to UV-induced skin damage.

**Figure 2:**
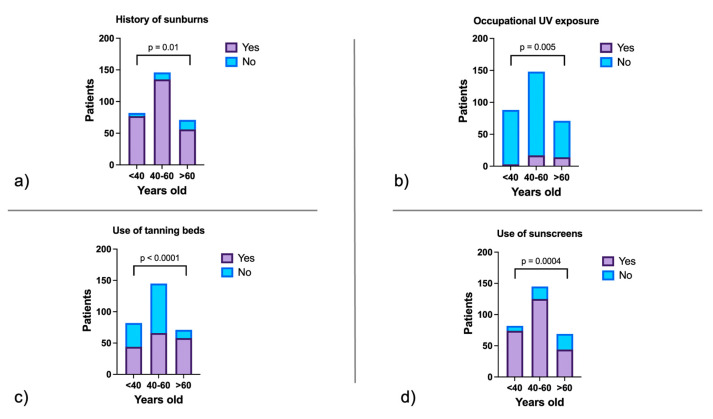
Distribution of melanoma patients according to UV exposure and prevention behaviors across age groups. (**a**) History of sunburns. (**b**) Occupational UV exposure. (**c**) Use of tanning beds. (**d**) Use of sunscreens. Older patients (>60 years) reported the highest levels of occupational and recreational UV exposure (19.7%, *p* = 0.005) and tanning bed use (81.7%, *p* < 0.0001), alongside significantly lower sunscreen use (35.2% reported no sunscreen use, *p* = 0.0004). In contrast, younger patients (<40 years) reported more frequent sunburns during both childhood and adulthood, while older patients reported more sunburns in adulthood (*p* = 0.01).

A significant portion of patients (59.2%) reported a history of sunburns during both childhood and adulthood, highlighting the cumulative impact of UV exposure on melanoma risk. Notably, 72.7% of patients reported consistent sunscreen use. Occupational UV exposure was documented in 10.5% of cases, underscoring the role of professional environments in melanoma risk (Figure 2).

Initial detection of melanoma lesions varied. Dermatologists were the first to identify suspicious lesions in 35.1% of cases, followed by patient self-detection in 33.9% of cases. Lesions were identified by friends or relatives in 7.8% of cases and by general practitioners or other specialists in 5.7% of cases. The detection pattern shifted for patients who developed a second melanoma, with dermatologists identifying the second lesion in 66.9% of cases, while patient self-detection decreased to 14.2%. Detection by friends or relatives and general practitioners was lower for subsequent melanomas.

Genetic testing was offered as part of routine clinical practice to patients who met the IMI eligibility criteria [19]. Patients who consented to testing received pre- and post-test genetic tele-counselling from the Cancer Genetics Unit in Genoa, which provided them with all necessary information. Of the study cohort, 55.9% (186 patients) met the criteria and consented to genetic testing. Of those tested, 4.3% (8/186 patients) had pathogenic (PV) or likely pathogenic variants (LPV) associated with melanoma susceptibility (only class 5 “pathogenic” and class 4 “likely pathogenic” variants were included in the analysis) [20,21]. Specifically, six patients carried a *CDKN2A* pathogenic variant (c.251A>T, c.71G>C, c.301G>T), one patient carried a *CDKN2A* likely pathogenic variant (c.458-1 G>C), and one patient carried a *POT1* pathogenic variant (c.258dupT).

### 3.1. Melanoma Characteristics

Age at melanoma diagnosis varied significantly across the cohort, with a median age of 48 years for the first melanoma and 58 years for the fourth melanoma. The age range was from 9 to 85 years. Histologically, superficial spreading melanoma was the most common subtype, accounting for 86.5% of first melanomas diagnosed. The mean interval between first and second melanoma diagnosis was 8.5 years (standard deviation: 7.3 years), with a median of 6.9 years and an interquartile range (IQR) of 2.6 to 11.5 years. The longest recorded interval was 37.4 years.

The mean Breslow thickness of the first melanomas was 0.9 mm, with a median of 0.4 mm (IQR: 0.2–0.8 mm) and a range of 0.0 to 20.0 mm. Subsequent melanomas showed progressively thinner lesions. Second melanomas had a mean Breslow thickness of 0.4 mm, with a median of 0.2 mm (IQR: 0.0–0.5 mm) and a maximum thickness of 8.4 mm. Third melanomas had a mean thickness of 0.3 mm (median: 0.3 mm; IQR: 0.0–0.5 mm), with a maximum of 1.4 mm. Fourth melanomas were the thinnest, with a mean thickness of 0.1 mm, a median of 0.0 mm (IQR: 0.0–0.2 mm), and a maximum of 0.3 mm.

TNM classification reflected these trends. In the first melanomas, 20.4% were classified as in situ (pTis) and 49.8% as pT1a. For second melanomas, the proportion of in situ cases increased to 43.8%, while 40.2% remained pT1a. A similar trend was observed for third melanomas, where 36.7% were pTis and 46.7% were pT1a. This shift towards a higher proportion of less invasive lesions over time suggests that subsequent melanomas are being detected at earlier stages.

The predominant growth pattern for the first melanomas was radial, accounting for 56.5% of cases. Regression was absent in 61.9% of cases, and ulceration was observed in 9.3%. Among all melanoma patients, the mean total number of nevi per participant was 110.3, with considerable individual variability. The mean number of atypical nevi was 3.4 (standard deviation: 7.8), with a median of 1 (IQR: 0.4), indicating significant variation in nevus burden and morphology between patients.

### 3.2. Analysis of Clinical and Demographic Characteristics by Age Group

The cohort was stratified into three groups according to age at first melanoma diagnosis: <40 years, 40–60 years, and >60 years (Table 2). The severity (Breslow thickness) of the first melanoma did not differ significantly between these groups (*p* = 0.31). However, significant age-related differences were observed in other clinical and demographic characteristics. Patients over 60 years of age had a significantly higher prevalence of multiple melanomas compared to younger age groups (*p* = 0.0002), with more than half presenting with multiple lesions. In contrast, familial melanoma was more common in younger patients, with 54.3% of patients younger than 40 years showing a familial pattern (Figure 1).

Ultraviolet (UV) exposure showed a clear age-related trend. Both occupational and recreational UV exposure were highest in the >60 age group (19.7%, *p* = 0.005), while sunscreen use was significantly lower in older patients. Among those aged >60 years, 35.2% reported no sunscreen use, compared to only 9.8% in the <40 group (*p* = 0.0004). In addition, tanning bed use was more common in older patients, with 81.7% of those >60 years reporting this practice (*p* < 0.0001). The history of sunburn varied by age group. Older patients reported more sunburns in adulthood, whereas younger patients had frequent sunburns in both childhood and adulthood (Figure 2).

Patients younger than 40 years had the highest total nevus count (mean: 139.6 vs. 111.4 vs. 74.1, *p* < 0.0001) and nevus density per square meter (*p* < 0.0001), both of which decreased progressively with age.

An interesting pattern emerged in the detection of suspicious lesions. Across all age groups, patients most often self-detected their first melanoma. However, for second melanomas in patients with multiple lesions, dermatologists played a more prominent role in identifying subsequent melanomas, especially in those aged 40–60 and >60 years (*p* = 0.001).

The distribution of melanoma subtypes remained consistent across all age groups, with superficial spreading melanoma being the most common type in all groups (*p* = 0.27).

## 4. Discussion

Analysis of patient characteristics based on age at first melanoma diagnosis reveals several key patterns that enhance our understanding of melanoma susceptibility and associated risk factors in different age groups. The mean age at diagnosis in this cohort was 48.5 years, which is consistent with the general median age of melanoma onset in the broader population [22].

Melanoma frequency varies by age, with a significantly higher prevalence of multiple melanoma cases in individuals over 60 years old. This suggests that age plays a key role in the development of multiple primary melanomas, probably due to cumulative UV exposure or genetic predisposition [23,24,25]. Case-control studies have shown a strong association between melanoma risk and the number of melanocytic nevi and sunburns during childhood [23,26]. Sunburns during childhood and adolescence significantly increase melanoma risk, suggesting that melanoma risk is primarily determined during childhood [27]. Occupational UV exposure and sunburns in adulthood are also important risk factors, especially in older individuals. This highlights the need for targeted prevention strategies for these groups [24]. UV exposure remains an important risk factor, with 59.2% of patients reporting sunburns in both childhood and adulthood. Although 72.7% of patients use high-SPF sunscreen, concerning behaviors persist, such as the use of tanning beds (37.5%) and reduced sunscreen application in older individuals. Additionally, while studies show a modest increase in melanoma risk associated with total or occupational sun exposure, intermittent sun exposure is more strongly linked to an increased melanoma risk [26]. The bimodal distribution of melanoma by age and site suggests two distinct mechanisms of UV-induced pathogenesis, supported by genetic studies [28]. Early-onset melanomas, which typically occur in middle-aged adults at sites with low cumulative sun damage (CSD), often harbor somatic *BRAF* or *NRAS* mutations, are associated with a higher number of nevi, and are primarily linked to intermittent UV exposure during childhood [28]. In contrast, late-onset melanomas, often of the lentigo maligna type, tend to occur at high-CSD sites, such as the head and neck. The genomic changes seen in high-CSD melanomas differ from those in low-CSD melanomas, highlighting the role of cumulative UV damage in their development, with a higher mutational burden and more frequent UV signature mutations [25]. Additionally, improper sunscreen use—such as inadequate application or prolonged sun exposure under the misconception of complete protection (the “sunscreen paradox”)—further exacerbates UV-related risks [29,30]. These findings, along with the high prevalence of occupational UV exposure in older populations, underscore the urgent need for targeted public health campaigns to promote sun safety and reduce harmful behaviors in all age groups [31,32].

Our cohort showed a slight predominance of females (54.9%), which is consistent with the literature indicating a higher incidence of melanoma in females, particularly in younger populations [33]. This trend is often attributed to behavioral factors, such as greater engagement in health care, more frequent skin self-examinations, and greater preventive behaviors compared to males [33]. Wojcik et al. highlight disparities in melanoma outcomes, particularly the survival disadvantage observed in adolescent and young adult (AYA) males. In older adults, this male survival disadvantage persists, suggesting the presence of additional biological or environmental factors [10]. Among AYAs, melanoma incidence is higher in females, partly due to tanning behavior. However, the increased risk of delayed detection in males highlights the need for targeted awareness and screening initiatives. In addition, AYAs diagnosed with melanoma are at higher risk for subsequent melanomas, especially if the first diagnosis occurs before the age of 30. Routine skin self-examination and annual clinical evaluation are critical to improving early detection and outcomes in this population [10].

Our study found that a significant proportion of patients (24.6%) had a university degree, suggesting a potential link between higher education and greater awareness of melanoma risks. This finding is consistent with existing literature, which shows that individuals with higher education levels are more likely to adopt preventive measures, such as using sunscreens with higher SPF values [34]. However, this group may also experience greater UV exposure, possibly due to increased recreational outdoor activities, which could partly explain the higher melanoma incidence observed among highly educated populations [35,36]. Despite this association, the impact of education on melanoma outcomes is complex. Some studies report no significant correlation between having a high school diploma and melanoma survival, while others have identified lower education levels as a predictor of poorer survival outcomes. Specifically, lower education has been linked to reduced overall survival (OS), potentially due to limited access to healthcare, lower health literacy, and delayed diagnoses. Similarly, residing in areas with lower education levels has been associated with worse melanoma outcomes, highlighting the role of social determinants of health in disease prognosis [35,37,38].

Marital status appears to be another important factor in melanoma outcomes. Studies indicate that unmarried individuals have reduced survival probabilities, while marriage acts as a protective factor. The benefits of marriage may go beyond the potential for early lesion detection by a spouse; they also include behavioral and psychosocial advantages. Spouses may encourage timely medical evaluations, promote protective behaviors such as sunscreen use, and offer emotional and logistical support during treatment, which can positively impact outcomes [34,38].

The development of multiple melanomas is well documented, and patients with a history of melanoma have a significantly higher risk of developing subsequent melanomas compared to the general population. In our cohort, the median interval between first and second melanoma was 6.9 years (IQR: 2.6–11.5 years), highlighting the persistent nature of melanoma risk. This variability underscores the complex interaction of genetic predisposition, environmental exposures, and follow-up practices and emphasizes the need for personalized, long-term surveillance strategies. The higher prevalence of multiple melanomas in older patients further emphasizes the importance of continued surveillance in this population [27]. A recent meta-analysis by Smith et al. identified several consistent risk factors for subsequent primary melanomas. Genetic predisposition, including pathogenic variants in *CDKN2A*, was strongly associated with an increased risk, along with a high total nevus count, particularly atypical nevi [32]. In our cohort, younger patients exhibited the highest total nevi counts and nevus density, suggesting an age-related trend in melanoma susceptibility linked to nevus burden. This finding is consistent with the existing literature, which emphasizes the importance of nevus burden as a significant risk factor [39,40]. People with more than 100 nevi are about seven times more likely to develop melanoma than those with fewer than 15 nevi [39]. This association is influenced not only by environmental factors such as UV exposure, but also by genetic predisposition. An inherited tendency to develop nevi can lead to familial clustering of melanoma cases, suggesting that genetic factors play a role in both nevus formation and melanoma susceptibility [39,40].

A strong family history of melanoma increases the likelihood of developing additional melanomas, emphasizing the need for targeted surveillance in genetically predisposed populations [32]. Environmental and phenotypic factors also play an important role. Light skin type and a high number of nevi are strongly associated with increased risk, while inadequate sun protection and high UV exposure continue to increase melanoma risk, especially in older patients. Smith et al. also reported that male sex, older age, and certain body sites (e.g., head and neck) are associated with increased melanoma risk, emphasizing the multifactorial nature of the disease [32].

Family history was identified as a key risk factor, with 59.5% of patients reporting a family history of melanoma and 16.5% reporting a family history of other cancers, particularly pancreatic (60%) and kidney cancer. These findings suggest potential shared genetic pathways and highlight the need for further research into the genetic links between melanoma and other malignancies [12,13,14,15,16]. Among patients with familial melanoma, 18.8% had a first-degree relative diagnosed with melanoma before the age of 60, highlighting the importance of rigorous screening in this high-risk group.

Genetic testing identified pathogenic/likely pathogenic variants in 4.3% of cases, primarily involving *CDKN2A*. Genetic counseling plays a critical role in the management of multiple and familial melanoma, especially in high-risk individuals. This process includes risk assessment, informed consent, disclosure of test results, and psychosocial support. It helps identify individuals at high genetic risk, those at intermediate risk due to multifactorial factors, and those at average risk. It also provides guidance to at-risk family members. Genetic counseling educates patients about hereditary melanoma syndromes, inheritance patterns, and the implications of genetic testing while also promoting preventive strategies such as UV protection and lifestyle changes [41,42,43]. Counselors also address the psychological impact of hereditary cancer risk, helping to reduce anxiety and provide emotional support. Even in families without detectable pathogenic variants, an increased risk of melanoma may persist due to other susceptibility genes and/or shared environmental factors. This underscores the importance of regular dermatologic screening for early detection. Referrals to melanoma genetic assessment units ensure comprehensive evaluation and personalized care, improving prevention, early diagnosis, and clinical outcomes for high-risk groups [41,42,43]. Genetic testing for melanoma predisposition should be performed using well-defined selection criteria and accompanied by thorough test interpretation and genetic counseling. Patients have to be informed that interpretation of test results can be complex and the potential impact on clinical management may be limited. Testing is recommended for individuals with at least a high probability of carrying a pathogenic variant, such as those from melanoma-prone families, families with melanoma-related cancers, or individuals with multiple primary melanomas. Leachman et al. proposed a practical framework for selecting candidates based on regional melanoma incidence and prevalence of genetic variants [15,43].

Advancements in genetic research have identified novel markers and interventions that address key gaps in melanoma care. Multigene panel testing, including genes like *CDKN2A*, *CDK4*, *BAP1*, *POT1*, *ACD*, and *TERF2IP,* enhances the detection of hereditary melanoma risk [14,17,44,45]. These genes play crucial roles in telomere maintenance, DNA repair and chromatin remodeling, which are critical pathways in melanoma genesis. For instance, *BAP1* variants are linked to a multi-tumor predisposition syndrome, while *POT1* variants disrupt telomere integrity, leading to genomic instability. Additionally, *MITF* (p.E318K variant) and pigmentation-related genes like *MC1R* influence melanoma susceptibility, particularly through UV sensitivity [14,17,44,45].

The low percentage of melanoma patients testing positive for germline pathogenic variants, even with expanded panels, reflects the complexity of the disease. This may be influenced by several factors, such as low or intermediate penetrance of genes (e.g., *MITF* and *ATM*), polygenic inheritance involving many low-risk genes not included in panels, regional founder mutations like *CDKN2A*, and the interplay of environmental factors such as sun exposure and genetic modifiers [14,17,44,45]. These findings underscore the need for revised assessment criteria, the discovery of new susceptibility genes, and a deeper understanding of the interactions between genetic, environmental, and lifestyle factors. To improve the identification of patients with genetic variants, future research should focus on expanding the knowledge of melanoma-associated genes while establishing robust clinical and epidemiologic criteria, such as number of affected relatives and age of disease onset, to optimize patient selection for genetic testing.

Innovative strategies are actively being developed to fill gaps in melanoma treatment, with nanotechnology emerging as a promising approach. Nanosystems serve as efficient drug carriers and light absorbers, enhancing the efficacy of existing treatments [46,47]. In addition, research into genetic markers, variants, and targeted therapies specific to melanoma subtypes is critical to advancing precision medicine [48]. Cutting-edge modalities such as neoantigen vaccines, adoptive cell transfer, microbiome-based therapies, and nanoparticle-based combination treatments are showing significant potential [49,50]. These advances are particularly important for improving outcomes in advanced melanoma or in patients with therapy-resistant disease, underscoring the need for continued research and integration of these novel interventions [49,50].

Tailoring interventions based on subtype-specific genetic alterations also holds potential for improving treatment outcomes [51,52]. Continued research on novel genetic markers remains essential for bridging gaps in melanoma care, advancing risk assessment, and optimizing management strategies.

## 5. Limitation

This study has several limitations that need to be considered. First, its retrospective nature may introduce biases inherent in data collection and analysis, potentially affecting the generalizability of the findings. In addition, not all patients underwent genetic testing, which may have limited the ability to fully assess genetic predisposition. The limited significance of the genetic findings underscores the need for larger cohorts to validate these results and provide a more comprehensive understanding of genetic contributions to melanoma.

Another limitation is the absence of a control group, such as patients with non-multiple or non-familial melanomas, or individuals without melanoma, which would have facilitated a more robust comparative analysis. Finally, although the sample size is substantial, it is not extensive. Nevertheless, the depth and breadth of information collected for each individual patient offer valuable insights into the disease, particularly for those with a higher burden of melanoma.

## 6. Conclusions

Melanoma can occur at any age, emphasizing the importance of early detection through consistent screening programs. Older patients (>60 years) exhibit a higher prevalence of multiple melanomas, which may be attributed to cumulative ultraviolet (UV) exposure and genetic predisposition. This underscores the necessity for prevention and monitoring strategies tailored to this age group. Sun exposure, frequent sunburns, and tanning bed use remain key risk factors, particularly among older individuals. Genetic testing identified pathogenic or likely pathogenic variants in 4.3% of patients, predominantly in the *CDKN2A* gene, highlighting the value of personalized management approaches. Additionally, family history and a high nevus burden are significant risk factors for multiple melanomas, emphasizing the critical need for intensive surveillance in high-risk populations.

This study underscores the complex interplay between genetic, environmental, and behavioral factors in the development and progression of melanoma. Future research should focus on further elucidating these mechanisms, particularly through longitudinal studies, to optimize prevention and monitoring strategies. Such advancements have the potential to improve clinical outcomes and guide public health initiatives aimed at reducing the burden of melanoma, especially in vulnerable populations. Looking ahead, integrating epidemiological and statistical data into computational algorithms powered by artificial intelligence holds significant promise. These technologies could facilitate early diagnosis, streamline triage processes, and empower patient self-detection. Moreover, they offer the potential to enhance risk stratification and support the creation of personalized follow-up pathways, ultimately improving outcomes for high-risk individuals.

## Figures and Tables

**Table 1:** Descriptive statistics of demographic and anamnestic characteristics of patients.

Characteristic	Total Sample
Patients, n (%)	333 (100.0)
Age at First Melanoma Diagnosis (years)	n = 323
	Mean (SD) = 48.5 (15.0)
	Median (IQR) = 48 (38–59)
	Min–Max = 9–85
Gender, n (%)	Female (F) = 183 (54.9)
	Male (M) = 150 (45.1)
Weight (kg)	n = 309
	Mean (SD) = 70.0 (13.6)
	Median (IQR) = 70 (60–80)
	Min–Max = 42–120
Height (cm)	n = 320
	Mean (SD) = 169.8 (8.7)
	Median (IQR) = 170 (164–176)
	Min–Max = 148–193
BMI	n = 307
	Mean (SD) = 24.1 (3.9)
	Median (IQR) = 23.9 (21.5–26.3)
	Min–Max = 17.2–39.1
Education Level, n (%)	University Diploma-Degree = 82 (24.6)
	Professional Diploma = 58 (17.4)
	High School = 96 (28.8)
	Lower Secondary = 57 (17.1)
	None-Primary License = 13 (3.9)
	Missing = 27 (8.1)
Marital Status, n (%)	Married = 220 (66.1)
	Single = 52 (15.6)
	Separated/Divorced = 21 (6.3)
	Widowed = 13 (3.9)
	Missing = 27 (8.1)
Melanoma Classification, n (%)	Multiple = 106 (31.8)
	Familial = 164 (49.2)
	Multiple and Familial = 63 (18.9)
Fitzpatrick Phenotype, n (%)	I = 23 (6.9)
	II = 171 (51.3)
	III = 112 (33.6)
	Missing = 27 (8.1)
Skin Color, n (%)	Light = 198 (59.5)
	Intermediate = 100 (30.0)
	Olive = 8 (2.4)
	Missing = 27 (8.1)
Hair Color at age 20, n (%)	Blonde = 68 (20.4)
	Light Brown = 114 (34.2)
	Dark Brown = 97 (29.1)
	Black = 15 (4.5)
	Red = 22 (6.1)
	Missing = 17 (5.1)
Eye Color, n (%)	Blue/Grey = 94 (28.2)
	Light Brown = 72 (21.6)
	Dark Brown = 73 (21.9)
	Black = 1 (0.3)
	Green = 76 (22.8)
	Missing = 17 (5.1)
Professional UV Exposure, n (%)	Yes = 35 (10.5)
	No = 280 (84.1)
	Missing = 18 (5.4)
History of Sunburns, n (%)	Yes (during adulthood) = 29 (8.7)
	Yes (during childhood) = 47 (14.1)
	Yes (both during childhood and adulthood) = 197 (59.2)
	No = 32 (9.6)
	Missing = 28 (8.4)
Sunscreen Usage, n (%)	SPF 30–50+ = 242 (72.7)
	SPF < 30 = 8 (2.4)
	No = 54 (16.2)
	Missing = 29 (8.7)
Use of Tanning Beds, n (%)	Yes = 125 (37.5)
	No = 179 (53.8)
	Missing = 29 (8.7)
Who first noticed suspicious lesion that later resulted in melanoma, n (%)	Other Specialist/GP = 19 (5.7)
	Friend/Other Relative = 26 (7.8)
	Spouse = 24 (7.2)
	Dermatologist = 117 (35.1)
	Patient Themselves = 113 (33.9)
	Missing = 34 (10.2)
Who first noticed second suspicious lesion that later resulted in melanoma, n (%) (*percentages calculated on patients with second melanoma*)	Other Specialist/GP = 4 (2.4)
	Friend/Other Relative = 1 (0.6)
	Spouse = 4 (2.4)
	Dermatologist = 113 (66.9)
	Patient Themselves = 24 (14.2)
	Missing = 22 (13.0)
Total Melanomas Experienced by Patient, n (%)	1 = 164 (49.2)
	2 = 139 (41.7)
	3 = 25 (7.5)
	4 = 4 (1.2)
	5 = 1 (0.3)
Genetics, n (%)	Conducted = 186/333 (55.9)
	PV/LPV = 8/186 (4.3)
	Not informative = 178/186 (95.7)
	Not Conducted = 147/333 (44.1)

If “n” differs from 333, it is due to missing data for certain variables. SD: standard deviation, IQR: interquartile range, BMI: body mass index, UV: ultraviolet light, SPF: sun protection factor, GP: general practitioner, PV: pathogenic variants, LPV: likely pathogenic variants.

**Table 2:** Patient characteristics stratified by age group at the diagnosis of the first melanoma.

		Age (Years)		*p*-Value
	<40	40–60	>60	
n	92 (100.0)	158 (100.0)	73 (100.0)	
Melanoma classification, n (%)				
Multiple	18 (19.6)	44 (27.8)	37 (50.7)	0.0002
Familial	50 (54.3)	85 (53.8)	29 (39.7)	
Multiple and familial	24 (26.1)	29 (18.3)	7 (9.6)	
Familial history of other related tumors *, n (%)				
Yes	18 (20.0)	29 (19.1)	7 (9.9)	0.17
No	72 (80.0)	123 (80.9)	64 (90.1)	
Fitzpatrick skin type, n (%)				
I	5 (6.0)	13 (8.9)	4 (5.6)	0.84
II	48 (57.8)	77 (52.7)	41 (57.7)	
III	30 (36.1)	56 (38.4)	26 (36.6)	
Occupational UV exposure, n (%)				
Yes	3 (3.4)	17 (11.5)	14 (19.7)	0.005
No	85 (96.6)	131 (88.5)	57 (80.3)	
History of sunburns, n (%)				
Yes (during adulthood)	8 (9.8)	11 (7.5)	10 (14.1)	0.01
Yes (during childhood)	14 (17.1)	27 (18.5)	6 (8.4)	
Yes (both childhood and adulthood)	55 (67.1)	97 (66.4)	40 (56.3)	
No	5 (6.1)	11 (7.5)	15 (21.1)	
Use of sunscreen, n (%)				
SPF 30–50+	71 (86.6)	122 (84.1)	44 (62.0)	0.0004
SPF < 30	3 (3.7)	3 (2.1)	2 (2.8)	
No	8 (9.8)	20 (13.8)	25 (35.2)	
Use of tanning beds, n (%)				
Yes	44 (53.7)	66 (45.2)	58 (81.7)	<0.0001
No	38 (46.3)	79 (54.5)	13 (18.3)	
Who first noticed the suspicious lesion later diagnosed as melanoma, n (%)				
Other specialist/GP	5 (6.5)	8 (5.5)	5 (7.0)	0.56
Friend/Other relative	10 (13.0)	10 (6.9)	5 (7.0)	
Spouse	3 (3.9)	15 (10.3)	5 (7.0)	
Dermatologist	26 (33.8)	59 (40.7)	30 (42.2)	
Patient themselves	33 (42.9)	53 (36.5)	26 (36.6)	
Who first noticed the second suspicious lesion later diagnosed as melanoma, n (%)				
Other specialist/GP	2 (6.1)	1 (1.5)	1 (2.4)	0.001
Friend/Other relative	0 (0.0)	0 (0.0)	2 (4.8)	
Spouse	1 (3.0)	1 (1.5)	2 (4.8)	
Dermatologist	18 (54.5)	55 (83.3)	35 (83.3)	
Patient themselves	12 (36.4)	9 (13.6)	2 (4.8)	
Total melanomas had by the patient, n (%)				
1 ^†^	50 (54.3)	85 (53.8)	29 (39.7)	0.24
2	38 (41.3)	64 (40.5)	37 (50.7)	
3	3 (3.3)	8 (5.1)	6 (8.2)	
4	0 (0.0)	1 (0.6)	1 (1.4)	
5	1 (1.1)	0 (0.0)	0 (0.0)	
Location, n (%)				
Head–neck	9 (10.0)	8 (5.1)	8 (11.0)	0.19
Upper limb	11 (12.2)	22 (14.0)	14 (19.2)	
Hand	0 (0.0)	0 (0.0)	4 (5.5)	
Chest	9 (10.0)	14 (8.9)	6 (8.2)	
Abdomen	3 (3.3)	9 (5.7)	27 (37.0)	
Back	24 (26.7)	64 (40.8)	0 (0.0)	
Gluteus	1 (1.1)	4 (2.5)	0 (0.0)	
Lower limb	31 (34.4)	34 (21.7)	13 (17.8)	
Foot	2 (2.2)	2 (1.3)	1 (1.4)	
Type, n (%)				
Superficial spreading	79 (89.8)	145 (92.9)	63 (96.3)	0.27
Acral lentiginous	1 (1.1)	0 (0.0)	0 (0.0)	
Choroidal/Ocular	0 (0.0)	0 (0.0)	0 (0.0)	
Lentigo maligna	2 (2.3)	3 (1.9)	3 (4.1)	
Nevoid	0 (0.0)	1 (0.6)	0 (0.0)	
Nodular	2 (2.3)	6 (3.8)	5 (6.8)	
Amelanotic nodular	1 (1.1)	1 (0.6)	1 (1.4)	
Spitzoid	3 (3.4)	0 (0.0)	1 (1.4)	
Growth pattern, n (%)				
Radial	46 (64.8)	95 (70.4)	47 (77.0)	0.31
Vertical	25 (35.2)	40 (29.6)	14 (22.9)	
Regression, n (%)				
Not identified	62 (74.7)	102 (73.4)	42 (63.6)	0.24
Present < 75%	16 (19.3)	33 (23.7)	18 (27.3)	
Present > 75%	5 (6.0)	4 (2.9)	6 (9.1)	
Ulceration, n (%)				
Present	9 (10.7)	13 (8.7)	9 (13.0)	0.60
Not identified	75 (89.3)	137 (91.3)	60 (87.0)	
Nevus-associated melanoma, n (%)				
Yes	28 (36.4)	51 (39.2)	11 (20.7)	0.05
No	49 (63.6)	79 (60.8)	42 (79.2)	
Number of mitoses				
n	78	141	68	0.02
Mean (SD)	0.5 (1.3)	1.1 (3.5)	1.7 (4.0)	
Median (IQR)	0.0 (0.0–0.9)	0.0 (0.0–0.9)	0.0 (0.0–1.0)	
Min–MAX	0.0–9.0	0.0–29.0	0.0–20.1	
Total nevi				
n	85	146	71	<0.0001
Mean (SD)	139.6 (77.0)	111.4 (77.8)	74.1 (69.6)	
Median (IQR)	135 (77–180)	97 (53–150)	55 (23–103)	
Min–MAX	3–397	1–463	0–280	
Total atypical nevi				
n	83	145	71	0.29
Mean (SD)	3.8 (6.8)	3.6 (9.4)	2.1 (3.7)	
Median (IQR)	1 (0–4)	1 (0–4)	0 (0–3)	
Min–MAX	0–39	0–99	0–19	
Percentage of atypical nevi				
n	83	145	69	0.44
Mean (SD)	2.1 (3.3)	2.8 (5.4)	3.3 (7.1)	
Median (IQR)	0.8 (0–3.4)	0.9 (0.0–3.1)	0.7 (0.0–3.8)	
Min–MAX	0.0–18.2	0.0–33.3	0.0–50.0	

If the sum of the values differs from 333, it is due to missing data for certain variables. SD: standard deviation, IQR: interquartile range, UV: ultraviolet light, SPF: sun protection factor, GP: general practitioner. * Pancreatic adenocarcinoma, uveal melanoma, pleural or peritoneal mesothelioma, renal neoplasms, or BAP1-inactivated melanocytic tumors. ^†^ Patients with a single melanoma diagnosis and a positive family history of melanoma.

## Data Availability

The raw data supporting the conclusions of this article will be made available by the authors on request.
